# Effect of micronutrients, rhizobium, salicylic acid, and effective microorganisms in plant growth and yield characteristics of green gram [*Vigna radiata* (L.) Wilczek] in Rupandehi, Nepal

**DOI:** 10.1016/j.heliyon.2024.e26821

**Published:** 2024-02-23

**Authors:** Jay Chaurasia, Balika Poudel, Taranand Mandal, Nobel Acharya, Vivek Ghimirey

**Affiliations:** a Department of Agricultural Extension and Rural Sociology, Institute of Agriculture and Animal Science, Tribhuvan University, Nepal; b Institute of Agriculture and Animal Science, Paklihawa Campus, Tribhuvan University, Nepal; c Department of Agricultural Extension and Rural Sociology, Agriculture and Forestry University, Nepal; d Department of Soil Science and Agri-engineering, Agriculture and Forestry University, Nepal

**Keywords:** Ammonium molybdate, Cobalt nitrate, Mungbean, Rhizobia, Salicylic acid

## Abstract

Nepal has a very diverse topography and ecosystem, with mountains in the north and lush plains in the south. Despite the diverse ecology, the production of green gram is still in its minority. This experiment was conducted to assess the effect of micronutrients (Zn, B, Mo, Co, and Mn), rhizobium, effective micro-organisms, and salicylic acid in plant growth, yield, and yield attributes of green gram [Vigna radiata (L.) Wilczek] in Rupandehi district of Nepal in March 2021. Pratikshya variety of green gram was used as a test crop. Different levels of ZnSO_4_ and Borax, Ammonium Molybdate, Cobalt Nitrate, MnSO_4_, effective microorganism, Mixture, Salicylic acid, and Rhizobia along with control constituted treatments. Various application methods were used for applying treatments including soil incorporation, seed priming, inoculation, and foliar application. The results revealed that leaf area index, number of branches, number of pods per plant, and yield were significantly different (P < 0.05). Among various treatments, the mixture was found most effective in the case of grain yield (1048.1 kg ha^−1^), stover yield (2472.7 kg ha^−1^), biological yield (3520.8 kg ha^−1^), harvest index (31.2%), and the number of primary branches (4.9). Likewise, the number of secondary branches (6.86) was better with Borax@10 kg ha^−1^. There was no significant difference in the plant height, number of pods per plant, pod length, pod weight, number of grains per pod, and test weight between different treatments. ZnSO4 @ 25 kg ha^−1^ was found to have an impact on the number of mature, immature, and total pods per plant. Overall, the study concluded that the mixture of all micronutrients performed better in terms of grain yield while zinc sulphate had great potential for plant growth parameters which could improve the farmers’ livelihood. It is recommended to conduct multi-location trials in a larger domain.

## 1 Introduction

Grain legumes are a significant part of the agricultural production of Nepal. It covers a major part in terms of area and production. Under the varying agroecological conditions, commonly grown grain legumes in Nepal are black gram, lentil, chickpea, cowpea, green gram, and soybean. Grain legumes position themselves in the fourth position in terms of cultivated area in Nepal. It covers 10.5% of the cultivated area, equal to 0.316 million hectares [1]. The total production of legumes was 382,987 metric tons in an area of 331,740 ha i.e. its productivity is 1.15 [2]. Grain legumes also called Pulses, are crops of the legume family (*Fabaceae*) cultivated specifically for their seeds for human food and animal feed. *Vigna radiata* is commonly known as green gram or mungbean.

Mung bean is a high-value crop with agricultural and nutritional benefits that contribute to food security and sustainable agriculture. It helps in fixing atmospheric nitrogen through a symbiosis between root nodules and soil micro-organisms promoting soil health [3]. Despite these benefits, the productivity of the green gram is recorded as low (0.5 ton ha^−1^) as compared to the potential yield of varieties (1.04 ton ha^−1^) in Nepal [4]. Low pulse yield is largely due to nutrient deficit [5]. Mostly macronutrients are applied and micronutrients are ignored. There is not enough domestic production of green gram where 90% of the current demand is fulfilled by the import of 5000 tons per year. This may be fixed by the proper nutrient application for the production whereas there is a lack of proper research and information on nutrient management and the seed quality of green grams [6]. To address the current scenario of lack of research and information about mung bean and its requirement of nutrients or additional applications, this experimentation was done to investigate the effect of micronutrients (Zinc, Boron, Cobalt, Molybdenum, Manganese), effective micro-organisms, rhizobium, and salicylic acid on plant growth, yield and yield attributes of mung bean.

Being a legume crop, green gram requires high micronutrients in the metabolic process and nitrogen fixation, but Nepalese farmers do not supply these nutrients. Mostly grown during the spring season (under moisture stress), micronutrients protect green grams from moisture stress and enhance nodulation and yield [7]. Previous studies also showed that rhizobium inoculation played a role in the increase of seed yield [3]. [8] highlighted in his findings that salicylic acid has a role in withstanding biotic and abiotic stress. It is considered that micronutrients Fe, Cu, Zn, Mn, Mo, and B help in the growth, yield, and grain quality of green gram [9,10]. Moreover, a study implied that the application of micronutrients is an effective method to enhance crop productivity [11]. In addition, it was found that EM should be applied with farmyard manure or the recommended dose of NPK fertilizers for mung bean to have better growth, yield, and nutrition [12].

Assuming there will be a significant difference in yield and seed quality of mung bean because of these treatments, the research was focused on exploring the possible changes in yield and yield parameters. The objective of this study was to provide the information and pave the way for future researchers emphasizing the nutrient requirements and other necessary applications to enhance the productivity of green grams. The study hypothesized that the application of different micronutrients, rhizobium, EM, and salicylic acid affects the growth, yield, and yield characteristics of mung bean. Besides, to motivate growers to produce such a promising crop, we decided to take the "*Pratikshya*" variety and bridge the gap between growers and the information required for production. Hence, the study will identify the best-performing micronutrients (Zn, B, Mn, Mo, Co), rhizobium, EM, and salicylic acid in mung bean and contribute to better decision-making for the application of these micronutrients, nutrient management, and sustainable production in smallholder farmers.

## 2 Materials and methods

### 2.1 Experimental site

A field experiment entitled “Effect of micronutrients, rhizobium, salicylic acid, and effective microorganisms in plant growth and yield characteristics of green gram [*Vigna radiata* (L.) Wilczek] in Rupandehi, Nepal” was conducted at the Agronomy farm of Institute of Agriculture and Animal Science, Paklihawa Campus, Tribhuvan University, Rupandehi to evaluate the performance of micronutrients in *Pratikshya* variety of green gram and identify suitable one for this agro-ecological zone. The research area is located in Sidharthnagar municipality at 27.5065° N, 83.4377° E.

### 2.2 Status of soil

Before running the experiment, a composite soil sample was taken and basic physicochemical properties were determined following standard protocols. Composite soil samples were randomly taken from different spots in a Z-manner using a tube auger from 25 cm depth to test the chemical properties of the experimental soil. The soil sample was then air-dried, grounded, and sieved through a 2 mm sieve. Finally, the soil sample was taken to the soil laboratory for analysis of chemical properties. The chemical properties of the soil of the research field are shown in Table 1. In the experimental field, the average pH of the soil was determined to be slightly acidic (pH 6.25) as illustrated in Table 1. One of the most frequently lacking elements in soil is boron. The pH of the soil affects Boron availability; acidic soils have the highest Boron availability. The primary determinant of Molybdenum (Mo) and Manganese (Mn) availability for plant absorption is the pH of the soil. The availability of Mo and Mn is typically increased when acid soils are limed [[13], [14], [15]] (see Table 2).

**Table 1:** Chemical properties of soil of experimental plot before sowing.

S.N.	Particulars	Observed value	Status
1.	Soil PH	6.25	Neutral
2.	Nitrogen(mg g^−1^)	1.52	High
3.	Available Phosphorous(μ g g^−1^)	10.06	Medium
4.	Available potassium(μg g^−1^)	52.32	High
5.	Organic matter (%)	1.36	Low
6.	Manganese(μg g^−1^)	0.76	Low
7.	Zinc(μg g^−1^)	0.75	Low
8.	Boron(μ g g^−1^)	2.16	Low

**Table 2:** Method of micronutrient application.

Treatments	Details	Dose	Method of application
T_1_	Control	–	–
T_2_	ZnSO_4_@10 kg ha^−1^	10 kg ha^−1^	Broadcasting in soil
T_3_	ZnSO_4_@25 kg ha^−1^	25 kg ha^−1^	Broadcasting in soil
T_4_	Borax@5 kg ha^−1^	5 kg ha^−1^	Broadcasting in soil
T_5_	Borax@10 kg ha^−1^	10 kg ha^−1^	Broadcasting in soil
T_6_	Ammonium Molybdate	5 g kg^−1^ of seed	Seed treatment
T_7_	Cobalt nitrate	1 g kg^−1^ of seed	Seed treatment
T_8_	Manganese Sulphate	5 g l^−1^	Foliar application
T_9_	EM	50 ml l^−1^	Spray in soil
T_10_	Mixture	Zn: 35 kg ha^−1^, B: 15 kg ha^−1^, Mo: 5g kg^−1^ ofseed, Co: 1 g kg^−1^ of seed, Mn: 5 g l^−1^; EM:50 ml l^−1^; Rhizobium(200 g kg^−1^of seed); Salicylic acid (1g@10lit of water)	Broadcasting in soil + seed treatment)
T_11_	Salicylic acid	1 g @ 10 lit of water	Foliar application
T_12_	Rhizobia	200 g kg^−1^ of seed	Seed treatment

### 2.3 Layout of the experimental field

**Fig. 1:**
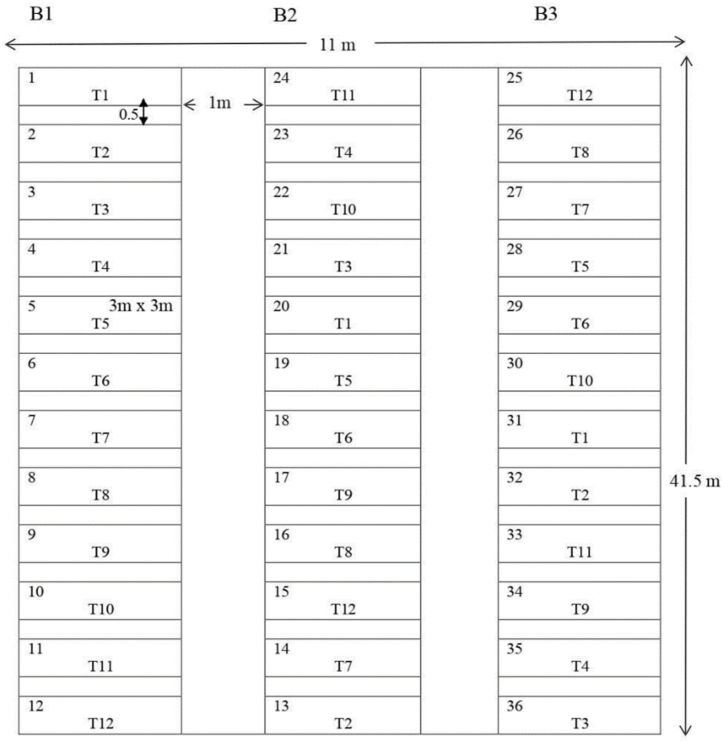
Layout of the experimental field.

Randomized Complete Block Design (RCBD) was used in the experiment. The size of the experimental unit was 9 m^2^ (3 m*3 m) and the total size of the experimental area was 456.5 m^2^ (11 m*41.5 m). The spacing between the blocks was 0.5 m and between experimental units within a block was 1 m. The total number of plots was 36 and in each plot, there were 12 rows of crops with 30 cm*10 cm spacing (Fig. 1).

### 2.4 Preparation of inoculum and micronutrient application

The experiment was laid out in a simple randomized complete block design (RCBD) with 12 treatments and 3 replications. Different micronutrients and *rhizobium* were considered as treatments. Zinc Sulphate (ZnSO_4_), Borax, and Effective Microorganisms (EM) were incorporated in the soil ^whereas Ammonium Molybdate^ at the rate(@) of 5g kg^−1^ of seed and Cobalt nitrate @1 g kg^−1^ of seed was seed treated. Foliar application of Manganese Sulphate and Salicylic acid was made at 20 and 30 days after sowing while other micronutrients were applied before sowing of the crops.

### 2.5 Data measurement and analyses

#### 2.5.1 Growth parameters

Growth parameters such as plant height, leaf area index, and nodulation were measured and studied during the experiment.

##### 2.5.1.1 Plant height

From each plot, 5 plants were selected randomly and the height of those plants was taken with the help of scale. It was measured from the base of the plant to the apex. The average plant height of 5 plants was calculated and expressed as the plant height of individual treatment.

##### 2.5.1.2 Leaf area index

Leaf area index (LAI) is a unit-less plant growth parameter that is obtained by dividing plant leaf area over plant land area [16]. From each plot, 5 sample plants were selected and the length and breadth of small, medium, and large leaves were taken with the help of scale. The breadth of the base, middle, and apex of each leaf was measured. The average breadth was calculated and the leaf area index was calculated by using the length and breadth of the leaves. The leaf area index can calculated using the following formula.

##### 2.5.1.3 Nodulation

Counting the total number of nodules on a plant is the simplest way to evaluate nodulation. The most common method of manually counting nodules is to remove them from the roots [17]. Five sample plants were taken and nodules were counted. The average number of nodules is expressed as the number of nodules of each treatment.

#### 2.5.2 Yield attributes and yield

##### 2.5.2.1 Number of pod-bearing branches per plant

Branches having pods were randomly selected to estimate the number of pod-bearing branches per plant. 5 sample plants were selected from each plot and then the average of five plants was recorded.

##### 2.5.2.2 Number of pods per plant

At harvest time, five randomly chosen plants from each plot were counted for the number of pods they produced; the mean number was then expressed for each plant.

##### 2.5.2.3 Pod characteristics

Pod characteristics such as the number of grains per pod, pod length, and pod weight were measured. For the number of grains per pod, pods were taken from randomly selected 5 plants from each plot and the number of grains per pod was counted, averaged, and recorded. Similarly, 5 sample plants were randomly selected for the pod length. The length of the pod was measured from the pod base to its tip with the help of a scale and then averaged and recorded. Also, the weight of pods m^−2^ was recorded and then the average pod weight was calculated and represented as the pod weight of each treatment.

##### 2.5.2.4 1000-Seed weight

The 1000-seed weight was taken after sun drying for 2–3 days. Then 1000 seeds were randomly selected and counted from each plot. After counting, the seeds were weighed using digital balance and then expressed in grams (g).

##### 2.5.2.5 Grain yield

Grain yield was calculated by harvesting 1 m^2^ from each experimental unit. After harvesting, grains were threshed, cleaned, weighed, and data was recorded. The grain yield was expressed in Kg m^−2^ which was later converted into Kg ha^−1^.

##### 2.5.2.6 Stover yield

Stover yield was also measured from 1 m^2^ from each experimental unit. The remaining biomasses were promptly weighed after the grain was threshed to determine the stover weight of each plot. The stover yield was expressed in Kg m^−2^ and then converted to Kgha^−1^.

##### 2.5.2.7 Biological yield

The biological yield was determined by weighing the stover, husk, and seed of 1 m^2^ area from each experimental plot. After calculating the biological yield in Kg m^−2^, it was converted to Kg ha^−1^.

##### 2.5.2.8 Harvest index

Harvest index (HI) was computed by using the formula suggested by Ref. [18] and recorded separately for each treatment. The harvest index is a unitless plant metric.

### 2.6 Statistical analysis

The collected data were tabulated in a Microsoft Excel worksheet. Growth parameters, nodulation, and yield parameters were measured and analyzed using a single factorial Randomized complete block design (RCBD). The Analysis of Variance (ANOVA) for all data was statistically analyzed using ADEL-R software. The Mean was separated by Duncan's Multiple Range Test (DMRT) at a 5% level of significance. The final result was interpreted with the help of necessary tables with related references.

## 3 Results

### 3.1 Growth parameters

#### 3.1.1 Plant height

Plant height was not significantly influenced by micronutrients at all dates of observations. However, the highest mean value for plant height was recorded at ZnSO4 @ 25 kg ha^−1^ which was 34.0 cm followed by Cobalt nitrate which was 33.7 cm. Previous reports have also indicated that this may be due to the role of zinc in auxin synthesis [19]. Similarly, the minimum plant height was recorded at Control which was 30.9 cm. The average plant height is shown in Table 3.

**Table 3:** Effect of micronutrients on plant height of green gram.

Treatment	15 DAS	30DAS	45DAS	60DAS
Control	7.5	9.2	22.4	30.9
ZnSO_4_ 10 kg ha^−1^	10.2	12.9	23.2	31.0
ZnSO_4_ 25 kg ha^−1^	11.5	13.8	26.3	34.0
Borax 5 kg ha^−1^	9.8	12.5	23.1	31.4
Borax 10 kg ha^−1^	9.5	12.1	24.8	32.1
Ammonium Molybdate	10.6	13.5	24.9	32.3
Cobalt Nitrate	10.3	12.7	25.7	33.7
Manganese Sulphate	10.3	12.6	25.1	31.3
EM	10.8	13.1	25.6	33.5
Mixture	10.6	13.2	24.4	32.6
Salicylic acid	9.6	11.8	22.8	32.7
Rhizobia	10.9	11.9	22.9	32.4
LSD	2.4	2.6	3.1	5.3
SEM	1.4	1.5	1.9	3.1
F test	NS	NS	NS	NS
F value	0.2	0.4	0.2	0.9
CV%	14.0	12.3	7.6	9.7
Grand Mean	10.1	12.4	24.3	32.3

Note: LSD: Least Significant Difference, CV: Coefficient of Variation, S E M: Standard error of mean NS: Non-significant (p > 0.05), DAS-Days after sowing.

#### 3.1.2 Leaf area index

Micronutrients significantly affected the leaf area index at 28 days after sowing (DAS) ^(P < 0.043). The highest leaf area index was recorded in Borax10^ kg ha^-1^ at ^35 DAS^ which was 0.6, followed by 42 DAS (0.5) as illustrated in Table 4. ^The minimum leaf area index (0.2) was recorded at 56^ DAS in Control.

**Table 4:** Effect of micronutrients on leaf area index at 7-day intervals for 56 DAS in green gram.

Treatments	Leaf area index							
	7DAS	14DAS	21DAS	28DAS	35DAS	42DAS	49DAS	56DAS
Control	0.02	0.03	0.05	0.4^ab^	0.4	0.4	0.3	0.2
ZnSO_4_ 10 kg ha^−1^	0.02	0.03	0.04	0.3^abcd^	0.4	0.4	0.4	0.3
ZnSO_4_ 25 kg ha^−1^	0.02	0.04	0.03	0.2^cd^	0.4	0.4	0.4	0.3
Borax 5 kg ha^−1^	0.02	0.04	0.06	0.2^cd^	0.4	0.4	0.3	0.3
Borax 10 kg ha^−1^	0.02	0.03	0.03	0.3^abc^	0.6	0.5	0.5	0.4
Ammonium Molybdate	0.02	0.03	0.04	0.3^abc^	0.4	0.4	0.4	0.3
Cobalt Nitrate	0.01	0.02	0.04	0.2^d^	0.5	0.5	0.5	0.4
Manganese Sulphate	0.02	0.05	0.06	0.2^cd^	0.4	0.4	0.4	0.3
EM	0.02	0.03	0.04	0.3^abcd^	0.4	0.4	0.4	0.3
Mixture	0.01	0.03	0.03	0.3^abcd^	0.5	0.5	0.4	0.4
Salicylic acid	0.02	0.06	0.06	0.4^a^	0.4	0.4	0.4	0.3
Rhizobia	0.01	0.04	0.05	0.1^bcd^	0.5	0.5	0.4	0.3
LSD	0.01	0.02	0.03	0.1	0.2	0.2	0.2	0.9
SEM	0.01	0.014	0.020	0.1	0.1	0.1	0.1	0.1
F test	NS	NS	NS	*	NS	NS	NS	NS
F value	0.16	0.10	0.51	0.1	0.9	0.4	0.3	0.4
CV%	35.33	31.26	35.37	19.6	31.4	30.4	31.7	33.7
Grand Mean	0.02	0.03	0.04	0.3	0.4	0.4	0.4	0.3

Note: *Ns*: Non-significant, DAS-Days after Sowing, *: significant (p < 0.05). Two different alphabetical notations denote significant differences between the respective means.

#### 3.1.3 Nodulation

The nodulation of green gram was found non-significant different among various treatments. The mixture has shown the maximum number of nodules (25.6) followed by rhizobia (24.6) with a grand mean of 17.6. The minimum number of nodules (10.0) was found in Cobalt nitrate followed by control (10.3). The average number of nodules per plant is shown in Table 5.

**Table 5:** Effect of micronutrients on the number of nodules per plant of a green gram.

Treatment	15DAS	30DAS	45DAS
Control	5.0	7.6	10.3
ZnSO_4_ 10 kg ha^−1^	6.3	8.7	12.6
ZnSO_4_ 25 kg ha^−1^	6.7	10.7	16.2
Borax 5 kg ha^−1^	8.3	13.4	19.2
Borax 10 kg ha^−1^	8.6	15.1	20.4
Ammonium Molybdate	11.3	17.0	23.6
Cobalt Nitrate	4.7	7.3	10.0
Manganese Sulphate	7.9	13.4	16.6
EM	7.0	10.7	15.0
Mixture	13.0	18.1	25.6
Salicylic acid	6.9	11.0	17.7
Rhizobia	12.0	17.5	24.6
LSD	1.3	1.3	2.3
SEM	0.8	0.8	1.4
F test	NS	NS	NS
F value	0.1	0.3	0.9
CV%	9.8	6.0	7.8
Grand Mean	8.1	12.5	17.6

Note: LSD: Least Significant Difference, CV: Coefficient of Variation, *Ns*: Non-Non-significant (p > 0.05), DAS-Days after sowing.

### 3.2 Yield attributes and yield

#### 3.2.1 Number of branches at harvest

The analysis of variance for primary and secondary branches showed significant differences among the treatments used in this experiment. The maximum average ^number of primary branches was observed in the mixture (4.9) whereas the least was^ observed in the control (3.9) with an overall mean value of 4.4. Similarly, the maximum ^average number of secondary branches was observed in Borax@10kg ha−1 (6.9),^ and the minimum was at control (5.2) with an overall mean value of 6.3 as illustrated in Table 6. This may be the result of increased growth characteristics.

**Table 6:** Effect of micronutrients on the number of branches of green gram.

Treatment	Number of branches	
	Primary branches	Secondary branches
Control	3.9^f^	5.2^g^
ZnSO_4_ 10 kg ha^−1^	4.1^e^	6.5^bcd^
ZnSO_4_ 25 kg ha^−1^	4.3^c^	6.8^ab^
Borax 5 kg ha^−1^	4.3^cd^	6.5^bcd^
Borax 10 kg ha^−1^	4.6^b^	6.9^a^
Ammonium Molybdate	4.7^b^	5.6^f^
Cobalt Nitrate	4.2^cde^	6.3^de^
Manganese Sulphate	4.7^b^	6.2^e^
EM	4.3^c^	6.5^bcd^
Mixture	4.9^a^	6.6^abc^
Salicylic acid	4.1^de^	6.3^cde^
Rhizobia	4.2^de^	6.2^e^
LSD	0.1	0.3
SEM	0.9	0.2
F test	*	*
F value	0.3	0.2
CV%	2.0	2.8
Grand Mean	4.4	6.3

Note: *: significant (p < 0.05). Two different alphabetical notations denote significant differences between the respective means.

#### 3.2.2 Number of pods per plant

There was a significant difference among the treatments in the number of mature pods per plant, the number of immature pods per plant, and the total number of pods per plant. However, the number of tender pods per plant did not show a significant effect. The maximum number of mature pods per plant (13.9), the number of immature pods per plant (4.3), and total pods per plant (20.3) were observed in ZnSO_4_ @ 25 kg ha^−1^. Likewise, the minimum number of mature pods per plant (10.4), the number of immature pods per plant (2.3), and the number of total pods per plant (14.9) were observed in Control (Table 7).

**Table 7:** Effect of micronutrients on the number of pods per plant of green gram.

Treatments	Number of mature pods per plant	Number of immature pods per plant	Number of tender pods per plant	Total pods per plant
Control	10.4^f^	2.3^c^	2.2	14.9^h^
ZnSO_4_ 10 kg ha^−1^	13.1^abc^	2.4^c^	2.5	18.1^cd^
ZnSO_4_ 25 kg ha^−1^	13.9^a^	4.3^a^	2.5	20.3^a^
Borax 5 kg ha^−1^	13.3^ab^	2.5^c^	1.7	17.5^def^
Borax 10 kg ha^−1^	13.7^a^	4.2^a^	1.4	19.3^b^
Ammonium Molybdate	13.5^a^	3.3^b^	1.5	18.3^c^
Cobalt Nitrate	12.1^de^	2.5^c^	2.8	17.4^ef^
Manganese Sulphate	11.7^e^	2.5^c^	2.0	16.2^g^
EM	12.6^cd^	3.2^b^	1.6	17.4^ef^
Mixture	12.8^bcd^	2.4^c^	2.6	17.8^cde^
Salicylic acid	12.2^de^	3.6^b^	2.1	17.9^cde^
Rhizobia	12.4^d^	2.4^c^	2.1	16.9^f^
LSD	0.6	0.4	0.3	0.7
SEM	0.4	0.3	0.2	0.4
F test	**	**	NS	**
F value	0.2	0.1	0.7	0.1
CV%	3.0	8.469	8.134	2.266
Grand Mean	12.6	3.0	2.1	17.7

Note: NS: Non-significant (p > 0.05), * *: highly significant (p < 0.01), Two different alphabetical notations denote significant differences between the respective means. The columns represented by the same letter (s) are not significantly different from each other.

#### 3.2.3 Pod characteristics

The analysis of variance for these traits indicated the presence of no significant differences among the treatments in pod length, pod weight, and the number of grains per pod. The average number of grains per pod ranges from 8.1 to 10.7 with an overall mean value of 9.2 (Table 8). Maximum average pod length (8.5 cm), pod weight (54.5g), and the number of grains per pod (10.7) were observed in the mixture. The minimum average pod length (7.7 cm), pod weight (31.8g), and the number of grains per pod (8.1) were observed in the control.

**Table 8:** Effect of micronutrients on pod characteristics of green gram.

**Treatment**	**Pod length(cm)**	**Pod weight(gm)**	**Number of grains per pod**	**1000-seed weight (gm)**
Control	7.7	31.8	8.1	35.3
ZnSO_4_ 10Kgha^−1^	7.9	48.6	9.2	44.3
ZnSO4 25 kg ha^−1^	8.1	50.2	8.8	39.9
Borax 5 kg ha^−1^	7.9	37.5	8.3	41.7
Borax 10 kg ha^−1^	7.9	35.1	9.0	38.3
Ammonium Molybdate	8.1	35.4	9.2	40.5
Cobalt Nitrate	7.9	40.8	10.0	36.9
Manganese Sulphate	8.4	44.8	9.9	45.0
EM	7.8	48.5	9.0	47.5
Mixture	8.5	54.5	10.7	56.3
Salicylic acid	8.1	37.4	9.3	45.1
Rhizobia	7.8	38.2	9.2	44.6
LSD	1.1	42.6	1.9	14.4
SEM	0.6	25.2	1.2	8.5
F test	NS	NS	NS	NS
F value	0.3	0.3	0.6	0.5
CV%	7.4	60.1	12.5	19.8
Grand Mean	8.1	41.9	9.2	43.1

Note: LSD: Least Significant Difference, CV: Coefficient of Variation, *Ns*: Non-significant.

#### 3.2.4 1000-Seed weight

Grain weight is an important quality attribute although this character is genetically controlled. The growing condition exerts considerable influence on its expression. There was no significant difference among various treatments in the case of 1000-seed weight. The ^mean value of 1000−seed^ weight among the treatment ranges from 35.3g to 56.3g with a grand mean value of 43.1g ^(^Table 8^). Among various^ treatments, the enhanced 1000 seed weight in green gram was seen by the application ^of a mixture (56.3g) followed by effective microorganisms (47.5g).^

#### 3.2.5 Grain yield

A significant difference was observed in grain yield among various treatments. The average mean value of grain yield among the treatment ranges from 459.6 kg ha^−1^ to 1048.1 kg ha^−1^ with a grand mean value of 861.8 kg ha^−1^ (Table 9). Among various treatments, the enhanced grain yield in green gram was seen by the application of mixture (1048.1 kg ha^−1^) followed by rhizobia (1005.3 kg ha^−1^).

**Table 9:** Effect of micronutrients on grain yield, stover yield, biological yield, and harvest index green gram.

Treatment	Yield and Yield attributing characters			
	Grain Yield (kg/ha)	Stover Yield (kg/ha)	Biological Yield (kg/ha)	Harvest Index (%)
Control	459.6^j^	1858.7^e^	2318.3^j^	19.8^e^
ZnSO_4_ 10 kg ha^−1^	764.7^i^	1966.3^g^	2731.3^i^	28.1^d^
ZnSO_4_ 25 kg ha^−1^	815.1^h^	1989.0^fg^	2804.1^h^	29.1^c^
Borax 5 kg ha^−1^	911.7^e^	2012.7^f^	2924.3^f^	31.2^a^
Borax 10 kg ha^−1^	941.6^d^	2074.3^e^	3015.9^e^	29.8^b^
Ammonium Molybdate	972.9^c^	2325.7^b^	3298.5^c^	29.5^bc^
Cobalt Nitrate	859.0^g^	2175.0^d^	3034.1^e^	28.3^d^
Manganese Sulphate	817.1^h^	2060.0^e^	2877.1^g^	28.4^d^
EM	889.7^f^	2288.3^c^	3178.0^d^	28.1^d^
Mixture	1048.1^a^	2472.7^a^	3520.8^a^	31.2^a^
Salicylic acid	856.6^g^	2176.7^d^	3033.3^e^	28.2^d^
Rhizobia	1005.3^b^	2348.7^b^	3354.0^b^	30.1^b^
LSD	18.5	31.3	41.6	0.5
SEM	10.9	18.5	24.6	0.3
F test	**	**	**	**
F value	0.6	0.4	0.7	0.2
CV%	1.3	0.9	0.8	1.1
Grand Mean	861.8	2145.7	3007.5	28.4

Note: LSD: Least Significant Difference, CV: Coefficient of Variation, * *: highly significant (p < 0.01). Two different alphabetical notations denote significant differences between the respective means.

#### 3.2.6 Stover yield

The analysis of variance for these traits indicated the presence of significant differences among the treatments in stover yield. The highest stover yield (2472.7 kg ha^−1^) was observed at mixture followed by *Rhizobia* (2348.7 kg ha^−1^) and the lowest yield (1858.7 kg ha^−1^) was observed at control with an overall mean of 2145.7 kg ha^−1^. The mean value for the stover yield of all treatments is shown in Table 9.

#### 3.2.7 Biological yield

Biological yield is the sum of grain yield and stover yield. A significant difference was observed among various treatments regarding biological yield. The maximum biological yield was obtained from the mixture (3520.8 kg ha^−1^) and the minimum was at control (2318.3 kg ha^−1^) with a grand mean of 3007.5 kg ha^−1^ as shown in Table 9.

#### 3.2.8 Harvest index

The amount of photosynthates moving to the plant's economic parts is determined by the harvest index. There was a significant difference in the case of harvest index among various treatments. The highest harvest index (31.3%) was recorded at mixture and the lowest harvest index (19.8%) was at control with an overall mean value of 28.5% (Table 9).

## 4 Discussion

### 4.1 Growth parameters

Auxin, which is derived from plant shoots, is the primary hormone that stimulates the elongation of the main axis and the appearance of lateral roots, and it also affects the growth of roots [20]. In addition, Zinc decreases the leaching of nutrients such as nitrates, improving the efficiency of nutrient usage [21]. Translocation of absorbed zinc content to various plant parts satisfies the need for optimal development of the plant [22]. Thus, the application of zinc and micronutrients has been reported to increase plant height as previously recorded [23,24]. Additionally, cobalt stimulates a variety of developmental processes, such as the elongation of stems and coleoptiles, the opening of hypocotyl hooks, and the expansion and development of leaf discs [25]. According to the previous findings, normal crop growth depends on a sufficient supply of micronutrients, however, the lack of these nutrients in the soil is a major problem in developing countries [26].

One of a crop's most crucial biophysical characteristics is the Leaf Area Index (LAI), which is used to determine the crop's growth stage, estimate biomass, and identify long-term water stress [27]. The leaf area index (LAI) is an essential crop growth metric because it plays a crucial role in capturing the PAR. The likelihood that solar radiation will reach leaves rather than land will increase with a larger leaf area index. The process of photosynthesis will turn this incident solar radiation into carbohydrates, which will ultimately affect crop biomass [28]. Boron is known to be a prominent factor in limiting pulse yield and has a significant impact on plant nutrition [28]. Both the synthesis of chlorophyll and the metabolism of carbohydrates ^are aided by it.^ Application of boron in crops has enhanced leaf area index at all crop growth stages. The grand mean of the leaf area index was recorded as 0.3 at 28DAS. This might be accounted for increased activities of meristematic tissues of the plant, increasing the number and size of the cells which ultimately increased the leaf area index of plants [29].

From Tables 5 and it has been depicted that rhizobium inoculation and the combination of rhizobium with micronutrients had a significant effect on the number of nodules as reported in Ref. [30]. The combined application of boron, molybdenum, and zinc has shown a significant increase in nodulation [31]. However, the application of cobalt nitrate causes a reduction in nodulation status i.e., nodules number and nodules weight [32].

### 4.2 Yield attributes and yield

Table 6 points out that the combination of micronutrients and borax has shown a significant effect on primary and secondary branches respectively. According to Ref. [33], boron plays a critical role in tissue differentiation and carbohydrate metabolism. It is also a component of cell membranes, necessary for cell division, and has a regulatory effect on other elements. Additionally, it is essential for the transfer of sugar in plants and the creation of new cells in meristematic tissue, enhancing all growth parameters [31]. described in their findings that the significant function that boron plays in plant metabolism and the movement of photosynthates from source to sink accounts for the increase in the number of secondary branches. Similarly, a significant increase in the number of branches per plant has been reported by the application of boron [19] and micronutrient mixture [34] in different crops. The average number of branches at harvest is shown in Table 6.

The reason for the phenomenon shown in Table 7 may be the formation of both pollen and stamen. Zinc sulphate administration promotes more pod-bearing branches by aiding in the development of both stamens and pollens, according to Ref. [35]. The enhancing effect of Zinc, Boron, and Molybdenum on mature pods per plant has been reported in green gram [26]. Application of boron, molybdenum, and manganese and their mixture were found to reduce the number of tender pods per plant at maturity [36]. Likewise, the micronutrients might have an enhancing role in grain setting, improving the number of grains per pod. The enhancing effect of the combination of all micronutrients was observed on different pod characteristics in green gram [26]. Among the micronutrients, the application of zinc, boron, cobalt, manganese, and their mixture was found to have a relatively better effect on pod weight and the number of grains per pod [37].

Considering the greater mobilization of photosynthates to the developing grain by the application of micronutrients, it might be the reason for the increase in seed weight. Several earlier researchers [26,38] reported similar effects in tomato, brinjal, french bean, and green gram respectively. Moreover, the grain yield per plant depends on the number of mature pods per plant, seeds per pod, and average grain weight which are considered to be the important component of the grain yield. The effect of any factor on yield character especially on yield components is integrated and expressed in grain yield [39]. All other treatments were found to have moderate to low effects in enhancing the grain yield of this crop. The result of the present investigation agrees with the findings of several researchers on several crops [[40], [41], [42]]. Application of the combination of micronutrients and rhizobium increases nitrogen content in the soil which leads to the increase in the grain yield. This may be the result of nitrogen's significant function as a structural element of cell components and a chemical that is metabolically active. In addition to being a structural element of enzymes, protoplasm, and chlorophyll, it also promotes the uptake of potassium and phosphorus. Consequently, enhanced nitrogen availability, markedly enhanced absorption, dry matter buildup, and nutrient translocation throughout the reproductive stage all improved yield parameters that influenced grain production [43].

The combination of micronutrients like zinc, boron, and molybdenum along with rhizobium is thought to have increased the yield of green grams because it improved growth, which in turn led to increased photosynthate and nutrient production and translocation, ultimately increasing grain and straw production. Similar results were found in the research on green gram [44]. The constant supply of nutrients, which in turn enhanced the leaf area and dry matter production, resulting in the maximum stover output, may be the cause of the addition in the stover yield caused by the various treatments. This is also responsible for the maximum uptake of nutrients during the crop's growth phase as mentioned in the findings [45].

The result obtained from this experiment is according to the past findings in several crops [39,41]. The mixture of different micronutrients, especially Zinc sulphate has a great influence on straw and grain yield. Similarly, rhizobium incorporating atmospheric nitrogen into the soil may be the cause of this result. Zinc sulphate at its optimal dose has been shown to dramatically increase straw and grain yield in chickpeas, which may be the cause of the increase in biological output [46]. According to Ref. [35], it could be because of the ideal zinc sulphate dosage, which had a major impact on the development of grains and vegetative growth in chickpeas, increasing the biological yield.

NPK content of green gram is increased by the integrated use of micronutrients and rhizobium inoculation which enhances the yield of green gram. Soil microorganisms can mobilize the unavailable forms of nutrient elements to available forms which increase crop productivity [47]. Finally, the addition of a mixture enhances the early vigor thus helping in better yield [48].

## 5 Conclusion

This study examined the response of various micronutrients, rhizobium, effective microorganisms, and salicylic acid in the *Pratikshya* variety of green gram, through a field experiment. The result demonstrated that among various treatments, the mixture of all micronutrients and other treatments had performed better in terms of yield and yield attributes. Plant height and the number of mature, immature, and total pods were best in the soil with the application of ZnSO_4_ @ 25 kg ha^−1^. On the other hand, the control was seen as least performing for most of the growth and yield parameters as the micronutrients were deficient. Notably, the maximum number of nodules was recorded in the mixture, followed by rhizobia. In summary, the co-application of micronutrients enhances the possibility of achieving improved plant growth, nodulation, a higher yields. Therefore, the adoption of micronutrients is deemed efficacious in augmenting the production of Green grams which will result in food security and, a rise in the living standards of smallholder farmers. However, to accurately recommend the application of micronutrients, rhizobium, em, and salicylic acid for the production of mung bean, further multi-seasonal and location trials with proper irrigation facilities and different varieties are recommended on a larger scale.

## Data availability statement

Data will be made available on reasonable request.

## CRediT authorship contribution statement

**Jay Chaurasia:** Writing – review & editing, Writing – original draft, Methodology, Formal analysis, Data curation, Conceptualization. **Balika Poudel:** Writing – review & editing, Writing – original draft, Validation, Supervision, Conceptualization. **Taranand Mandal:** Writing – review & editing. **Nobel Acharya:** Writing – review & editing. **Vivek Ghimirey:** Writing – review & editing.

## Declaration of competing interest

The authors declare that they have no known competing financial interests or personal relationships that could have appeared to influence the work reported in this paper.
